# Best videos and reviewer of the year selection 2018

**DOI:** 10.1590/S1677-5538.IBJU.2019.01.02

**Published:** 2019

**Affiliations:** 1 Section Editor, International Brazilian Journal of Urology Senior Member; 2 Department of GU Oncology, Assistant Chief of Surgical Services Moffitt Cancer Center

One of my distinct honors as the editor of the video section is to reflect on the prior year and to highlight some of our major accomplishments and contributions, with once again our submitting authors highlighting the incredible surgical talents and innovation being adopted across the world. In this regard, I am pleased to once again share with you the selection of the three best videos of the year, with the criteria for selection being the accepted videos that most vividly depict the creative approaches to surgery within our field, with the ultimate goal of optimizing patient related outcomes. In this regard, I am pleased to announce the 1st prize selection being the video by Luciano Favorito et al. from the Hospital Federal de Lagoa in Rio de Janeiro, Brazil entitled “Double inlay plus ventral onlay buccal mucosa graft for simultaneous penile and bulbar urethral stricture” (available at. http://www.intbrazjurol.com.br/pdf/vol44n04/Favorito_838_839.pdf). The authors beautifully depict how long multifocal (3.5 cm and 3 cm, respectively) penile and bulbar can be approached using a ventral onlay buccal graft technique in a very successful manner. The authors highlight that although a number of surgical options for caring for such patients exists, the present approach is very well suited for managing such complex urethral reconstructive cases. Our selection for the 2nd prize is awarded to the video by Wilson Lin et al. from Yeshiva University Albert Einstein College of Medicine in New York, USA entitled “Addressing the challenges of reoperative robotic-assisted sacrocolpopexy” (available at. http://www.intbrazjurol.com.br/pdf/vol44n06/Lin_1263_1264.pdf). The authors highlight several important surgical concepts in this video including that minimally invasive robotic surgery remains a feasible surgical option in patients having undergone multiple abdominal and pelvic surgeries in the setting of pelvic organ prolapse. Furthermore, there are some very useful technical points and considerations that are disseminated in this video and would encourage surgeons tackling such cases to review and possible adopt some of these surgical refinements into their armamentarium. The 3rd prize for best video of the year goes to the video entitled “Step by step male to female transsexual surgery” (available at. http://www.intbrazjurol.com.br/pdf/vol44n02/Silva_407_408.pdf) by Rodrigo Uliano Moser da Silva et al. from Hospital de Clinicas de Porte Alegre from Brazil. The very skilled surgeons within this group have performed 174 such procedures as is highlighted and artfully depicted in the respective sequential technical steps that are encompassed within such complex surgical procedures. These are surgical procedures that unquestionably should be performed at a few select centers across the world and those programs entertaining the development or refinement of their transsexual surgical program should consider benchmarking this video as the reference on how this surgery can be accomplished in an optimal manner in terms of surgical outcomes and patient satisfaction.

Lastly, I would like to announce a new selection as part of this annual editorial which is to select the reviewer of the year for our section. Before doing so, I would like to acknowledge and recognize the meaningful contributions of all our reviewers who have meticulously reviewed all video submissions and resubmissions in a timely manner making thoughtful recommendations and selecting only those deemed of greatest clinical merit for inclusion within our journal. In this regard, I am pleased to announce that Dr. Trushar Patel, assistant professor of urology and program director, department of urology, University of South Florida as our selection for reviewer of the year. Trushar is not only an exceptionally skilled robotic surgeon conducting complex oncologic and reconstructive cases but has done so while being a very dedicated reviewer for the video section over the past several years and always providing great insight and practical viewpoints in his video reviews. Very much appreciate all of his work and assistance over the prior years and congratulate him on this recognition.

Finally, I would like to thank all of our readers and contributors for their limitless efforts to continually improve surgical techniques and approaches. May this be a very happy and healthy holiday period for all of you and your families, with our very best wishes in 2019!
